# Linked Exposures Across Databases: an exposure common data elements aggregation framework to facilitate clinical exposure review

**DOI:** 10.3389/fpubh.2024.1408222

**Published:** 2024-06-28

**Authors:** Immanuel B. H. Samuel, Kamila Pollin, Sherri Tschida, Michelle Kennedy Prisco, Calvin Lu, Alan Powell, Jessica Mefford, Jamie Lee, Teresa Dupriest, Robert Forsten, Jose Ortiz, John Barrett, Matthew Reinhard, Michelle Costanzo

**Affiliations:** ^1^The Henry M. Jackson Foundation for the Advancement of Military Medicine Inc., Bethesda, MD, United States; ^2^The War Related Illness and Injury Study Center, Washington, DC, United States; ^3^Complex Exposure Threats Center, Department of Veterans Affairs, Washington, DC, United States; ^4^Explosive Ordnance Disposal Information Management System (EODIMS), Air Force Civil Engineer Center, Functional Management Office (FMO) (AFCEC/CBS), Joint Base San Antonio, San Antonio, TX, United States; ^5^Department of Psychiatry, Uniformed Services University, Bethesda, MD, United States; ^6^Department of Medicine, Uniformed Services University, Bethesda, MD, United States; ^7^Georgetown University Medical School, Washington, DC, United States

**Keywords:** military exposures, data aggregation, exposure model, dose, exposure common data elements

## Abstract

Understanding the health outcomes of military exposures is of critical importance for Veterans, their health care team, and national leaders. Approximately 43% of Veterans report military exposure concerns to their VA providers. Understanding the causal influences of environmental exposures on health is a complex exposure science task and often requires interpreting multiple data sources; particularly when exposure pathways and multi-exposure interactions are ill-defined, as is the case for complex and emerging military service exposures. Thus, there is a need to standardize clinically meaningful exposure metrics from different data sources to guide clinicians and researchers with a consistent model for investigating and communicating exposure risk profiles. The Linked Exposures Across Databases (LEAD) framework provides a unifying model for characterizing exposures from different exposure databases with a focus on providing clinically relevant exposure metrics. Application of LEAD is demonstrated through comparison of different military exposure data sources: Veteran Military Occupational and Environmental Exposure Assessment Tool (VMOAT), Individual Longitudinal Exposure Record (ILER) database, and a military incident report database, the Explosive Ordnance Disposal Information Management System (EODIMS). This cohesive method for evaluating military exposures leverages established information with new sources of data and has the potential to influence how military exposure data is integrated into exposure health care and investigational models.

## Introduction

The health implications of military exposures are a major concern for clinicians and researchers in the field of Veteran healthcare, with 43% of Veterans expressing toxic exposure concerns to their Veterans Affairs (VA) healthcare providers (1). The recent passing of the PACT Act in 2022 expanded VA care and benefits while increasing presumptive health conditions for various military deployments and exposures. This surge in interest for military toxic exposures necessitates the integration of appropriate data to support investigations between exposures and health outcomes. Capturing the exposome, which measures the multifaceted relationships between environment, behavior, biology, and disease over time, is essential to this understanding (2) as exposures do not cease after military service. Utilizing an exposome model to understand military toxic exposures is crucial because it considers the totality of environmental influences on individuals across their lifetime (3). Similar needs are present in environmental health surveillance programs when integrating existing data. Recently, the United Kingdom completed pilot programs for data integration in communities with high-quality exposure data and paired this data with outcomes in their National Health System (4, 5). Also, current needs demand an expanded Environmental and Public Health Tracking (EPHT) system to emphasize the need of merging, integrating and interpreting exposure data and relating to health outcomes (6, 7). The proposed LEAD framework aims to support efforts to facilitate a unified exposure tracking methodology and help to further understand the exposome.

## Linked Exposures Across Databases (LEAD) framework

The LEAD framework unifies diverse exposure sources using common data elements, addressing gaps in sourcing and characterization. Focusing on clinical utility, it develops health applications for siloed sources like military records and incident reports. The LEAD method calculates total exposure dosage as a function of intensity and duration. This method of dosage estimation is used across fields such as radiation exposure capture (8), toxicity research (9) and the military to calculate blast over-pressure as the product of pressure over a set period (10, 11). However, such applications typically focus on specific exposures. LEAD’s methodology employs general definitions of Exposure Common Data Elements (ExCDE), enabling comprehensive exposure characterization across various sources while utilizing existing methods and shaping new approaches. This integration facilitates the translation of individual and population-level exposure data for clinical purposes and fosters toxic exposure surveillance and research applications.

While quantitative measures of exposure intensity (e.g., Pascals for blast pressure, micrograms for chemical exposure) and time of exposure (measured in seconds) using sensors are the most objective and quantifiable form of measurement, such information is rarely available at the individual level for Veterans concerned about toxic military exposures. Therefore, the LEAD framework aims to expand the application of intensity and time-based dosage estimation to large sets of qualitative and subjective data. Additionally, potential moderating factors such as protective controls are considered since these factors could moderate exposure-related outcomes.

LEAD characterizes exposure using the following exposure common data elementsExCDEs:


$$\mathrm{Exposure}\;\mathrm{Dose}\sim f\left(\mathrm{Intensity}\times \mathrm{Time}\;|\;\mathrm{Moderators}\right)$$

$$\mathrm{Intensity}\sim f\left(\mathrm{Route},\mathrm{Proximity},\mathrm{Symptoms}\right)$$

$$\mathrm{Time}\sim f\left(\mathrm{Duration},\mathrm{Frequency},\mathrm{Period}\right)$$

$$\mathrm{Moderators}\sim f\left(\mathrm{Environmental}\;\mathrm{Controls},\;\mathrm{Personal}\;\mathrm{Protective}\;\mathrm{Controls}\right)$$

**Figure 1:**
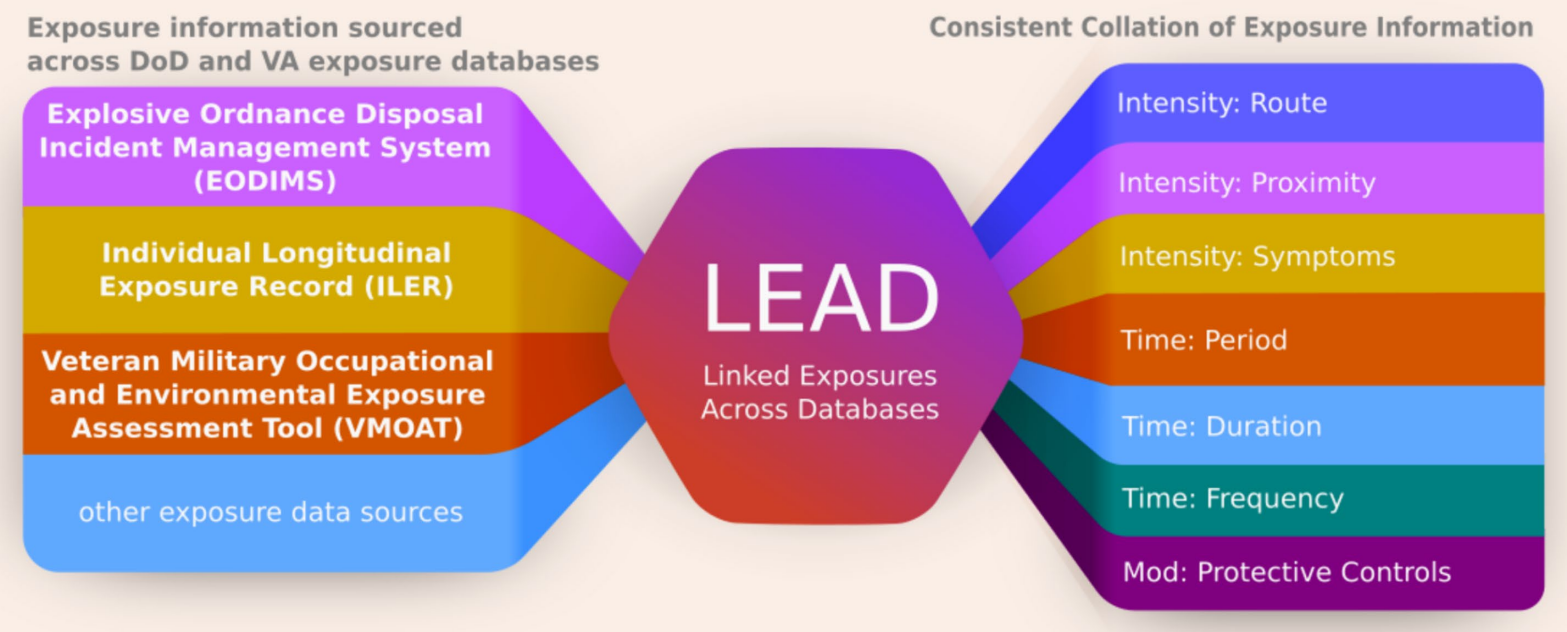
The LEAD Framework defines exposure common data elements to enable exposure information collation from a wide range of different databases and obtain a consistent log of exposures. This unform representation of exposures facilitates easy and consistent interpretation to help guide clinical care and research.

The LEAD framework allows for estimating aggregate exposure dosages using proxy measures of intensity and duration, along with factors that may influence these effects (Figure 1). The goal of the LEAD framework is to establish consistent parameters for characterizing exposures. However, like clinical practice where some features hold more weight due to their perceived impact on outcomes, the exposure variables also need to be weighted when evaluating exposure dose. This report provides expert-informed weights for specific variables, demonstrating a practical example of exposure dose estimation. Future analysis with health outcomes data can employ risk-modeling methods (e.g., Cox-Proportion Hazards Model) to assign empirical weights.

To illustrate how the LEAD framework can be used to consolidate exposure data into a unifiable metric, this report will present information from three different exposure data sources relevant to a cohort of Explosive Ordnance Disposal (EOD) Veterans, a complex and very high environmental exposure military occupation. We provide sample data extraction of exposure information from multiple databases and their collation in Table 1. Further, examples of how the data can be used to compare high and low blast exposure using a common scoring methodology is provided in Supplementary Tables S1–S3.

**Table 1:** Using the LEAD framework, exposures are characterized according to its route, proximity and symptoms at the time of exposure which reflects exposure intensity as well as the duration, frequency and period of the exposure event which reflects the exposure’s temporal characteristics.

1. Exposure collation		Intensity			Time			Moderators
Source	Exposure	Route	Proximity	Symptoms	Period	Duration	Frequency	Protective controls
EODIMS	Blast	Impact	10 feet	Lost consciousness	03/01/1999–03/02/1999	4 h	1 event	Suit
EODIMS	Blast/50lbs NEW	Impact	>150 m	None	07/01/2000–07/02/2000	< 2 min	1 event	Bunker
EODIMS	Blast/<5 lb. NEW	Impact	< 15 m	n/a	11/01/2000–11/02/2000	4 h	1 event	Safe area
VMOAT	Blast	Impact	10 feet	Lost consciousness	03/01/1999–03/02/1999	4 h	Frequent	Suit
ILER-PDHA	Vehicle accident	Impact	n/a	Neck/back injury; LOC <5 min; Disorientation	02/11/2000–02/11/2000	n/a	1 event	Seat belt
ILER-PDHA	Doxycycline (Vibramycin®)	ingestion	n/a	n/a	02/01/2000–11/01/2000	6 months	daily	n/a
ILER-PDHR-A	Sand/Dust	Inhalation	n/a	Coughing, difficulty breathing	04/01/2000–01/01/2001	10 months	n/a	n/a
ILER-EIR	Fuel	Skin, inhalation	10 feet	Rash	03/01/1999–03/02/1999	3 h	Less frequent	Mask, ventilation
ILER-POEMS	Food/water borne disease	Ingestion	n/a	High risk: bacterial diarrhea, hepatitis A, typhoid fever Moderate risk: diarrhea-cholera, diarrhea-protozoal, brucellosis and hepatitis E	04/01/2000–01/01/2001	daily	less frequent	Hepatitis A and typhoid vaccination Food/water consumption only from approved sources

Additionally protective controls used at the time of exposure is also recorded which may moderate the effects of exposure on health outcomes. Transforming exposure information into a consistent format as demonstrated in this table enables faster parsing of exposure information and reduces context switching which is necessary when reviewing exposure data across different databases.

Data sources include:

- Explosive Ordnance Disposal Information Management System (EODIMS): The EODIMS System is an operations specific Classified and Unclassified program for record incident reporting with discoverable data managed by the Air Force supporting joint service EOD units. Focused use of these records for clinically relevant exposure data enables access to the immediate post-exposure reports that are less affected by recall bias and may represent direct evidence of a possible hazardous exposure.
- Veteran Military Occupational and Environmental Exposure Assessment Tool (VMOAT): The VMOAT is a self-reported, structured, and lifespan-based comprehensive assessment of occupational exposures sustained during military service as well as during non-uniformed military and civilian work periods. The VMOAT includes a demographic information section, a lifespan-based occupational and environmental history section, and a comprehensive exposures section based on evidence-based exposure categories such as chemical, physical, injuries, biological, and psychological (12, 13).
- Individual Longitudinal Exposure Record (ILER): The ILER is an individual, electronic record of exposures for each service member and Veteran. ILER contains information from other sources including deployment dates and locations, all-hazard occupation data, environmental hazards, objective monitoring, medical encounter information and medical concerns regarding possible exposures. ILER aims to deliver capabilities and improvements in health care, benefits, collaborations between VA, DoD, Congress, beneficiaries, and other stakeholders (such as Veterans Service Organizations), as well as research and integration of exposure data from VA’s environmental health registries (14).

## Application of the LEAD to collate exposure variables across exposure databases

Integrating data into exposure variables to be analyzed when determining exposure dose across various hazard categories is a primary function of LEAD. Exposures are categorized according to domains: chemical, biological, physical, ergonomic/injury, and psychological hazards, as defined by the Department of Labor’s Occupational Safety and Health Administration (15). A description of each of these variables is detailed, along with examples of exposure information available across EODIMS, VMOAT and ILER.

### Intensity

Exposure intensity can be directly estimated by assessing the amount or concentration of hazardous substances that an individual comes into contact. In most cases, intensity exposure characterization occurs retrospectively with limited quantitative exposure data. In such cases, indirect measures of exposure or proxies for intensity such as routes of exposure, proximity to exposure source, and symptoms at the time of the exposure event are the only means of assessing intensity. Such indirect estimates supplement objective information that is often not available.

### Route

The route of exposure refers to how a substance enters the body. The EODIMS database does not contain extensive documentation of exposures routes, but routes may be inferred from the incident type and specific exposures reported. For example, an incident report documenting post-blast aerosolized particulate matter may be inferred to have inhalation and skin contact as possible exposure routes. The VMOAT1.0 categorizes exposure routes into the following categories: inhalation, ingestion, skin/eye contact, injection. Psychological exposures routes are categorized as experiencing, seeing, and/or hearing. ILER contains location and group-level exposure records that may be probabilistically associated with chemical (e.g., burn pits) and biological (e.g., infections) exposures.

### Proximity

The proximity to the exposure source is directly related to the intensity of exposure, as individuals who are closer to a hazard will generally experience higher exposure intensities and, subsequently, greater doses. Data from EODIMS can provide valuable information regarding proximity, including distances between safe areas and incident sites, as well as frequency of travel between these locations and others involved in the documented response. Although VMOAT1.0 did not estimate proximity, VMOAT2.0 aims to assess proximity to exposure more effectively. Additionally, proximity estimates can be obtained from ILER sources such as VA registries; however, these data are limited in scope and depth of information collected.

### Symptoms at the time of exposure

Presence of medical signs and symptoms following exposure may suggest higher exposure intensities. While the purpose of LEAD is not to assess health outcomes, assessing the presence of health changes immediately following exposure can be used as a proxy for estimating exposure intensity especially when objective exposure intensity data is unavailable. We note here that the absence of symptoms or the lack of documentation of symptoms should not be interpreted as a lack of exposure, as devastating health consequences may occur years after exposures (e.g., mesothelioma in asbestos workers) (16–18). While EODIMS documents capture immediate health effects associated with each exposure incident, such capture is neither consistent nor uniformly documented. ILER does contain limited records that ask subjective deployment health questions through DoD’s Post Deployment Health Reassessment (PDHRA) such as: “Were you wounded, injured, assaulted or otherwise hurt during your deployment?”

### Time

The timing of exposure is important and can be directly estimated with a variety of approaches, ranging from sensors with high sampling rates to subjective reports. Data at the individual level though is sparse and often requires interpreting subjective narratives of exposures that are incomplete and vary from person to person (for example, some individuals recall in detail the timing of an exposure whereas others will report a general time during a deployment). Duration and frequency of an exposure can be used to estimate exposure timing.

### Duration and frequency

The duration of exposure refers to how long someone is exposed to a substance or hazard. The longer the duration, such as noise (19), or long-term bio accumulation of Polyfluoroalkyl substances (20, 21), generally the greater the total dose and risk of adverse outcomes. Similarly, frequent repeated exposures even at smaller intensities, such as repeated blast exposures, can lead to chronic health effects (22). The assessment of duration and frequency in databases can be inconsistent due to the lack of standardized measures. Databases like EODIMS provide detailed time and frequency estimates such as start and completion times, while others like VMOAT measure ‘duration’ in terms of hours per day and ‘frequency’ in events within a specific time frame. ILER does not have distinct duration and frequency estimates for most cases, but some exposure Registry assessments consider the number of hours or days an individual may have been exposed in a typical day or month (e.g., airborne hazards including burn pits, fumes, dust, or other similar exposures).

### Period or the time of exposure

The exposure period is associated with occupational history or military deployments. The start and end dates for occupational periods are assessed either by asking for the start and end times for exposures in questionnaires, as done in VMOAT, or through administrative records contained in the ILER or EODIMS. Time of exposure and date of birth can be combined to estimate (i) age at exposure, (ii) time since exposure, and (iii) cohort effects across military eras which are key exposure factors that may affect health outcomes.

### Moderators

Hazard controls play an important role in moderating health risks associated with occupational exposure (23). While individual factors that affect exposure tolerance (24), are important moderating factors, they are not assessed broadly. Given the growing body of literature on the exposome and potential health outcomes (25, 26) it is important that exposure assessments incorporate these contributing factors as exposure science develops.

### Environmental and personal protective controls

The National Institute of Occupational Safety and Health (NIOSH) hierarchy of controls (27) has been developed to control worker exposures, reduce or remove hazards, and reduce risk of illness or injury. When elimination or substitution (i.e., most effective on the hierarchy) of the hazard is not possible, engineering controls such as ventilation systems, administrative controls of rotating work schedules and Personal Protective Equipment (PPE) may reduce cumulative exposures (28). The EODIMS records hazard data, personnel hours, equipment, disposition, and protective controls for many operational and training events. The VMOAT also asks hierarchy of control and PPE questions on its subjective exposure questionnaire. ILER documents Hazard Controls in Defense Occupational and Environmental Health Readiness System (DOEHRS) Industrial Hygiene (IH) reports, but these reports are usually done on a cohort level as opposed to the individual level.

### Sample LEAD framework exposure aggregation process

Drawing from the components of the LEAD framework, Table 1 illustrates the process of collating these exposure components across multiple sources into a consistent format. Additionally, Supplementary Table S1 illustrates how blast information can be compared to identify high- and low-level exposure using a simulated data based on typical EOD exposure concerns. Moreover, a translation layer can be used to reduce incompatible scoring between sources and improve consistency and interpretability when estimating exposure dose rates (Supplementary Tables S2, S3).

## Discussion

The Existing exposure assessment tools have limited scope, are inconsistently used, and often do not capture metrics that result in meaningful data relevant to clinical and research care. The LEAD framework outlines a consistent method of exposure information aggregation with simplified, exposure common data elements. It aims to improve exposure profiles by offering a standardized template for integrating exposure information across various sources to formulate exposure risk metrics that are easier for clinicians and researchers alike to understand, and integrate this information into Veterans’ care. Thus, the LEAD framework promotes consistency in exposure risk communication and interpretation of exposure-related health risks across clinical settings. These efforts aim to advance military exposure science and support exposure-informed clinical care for all Veterans.

Specifically, this framework provides the foundation to summarize exposures that are relevant for clinical care. Ongoing efforts are aimed at condensing detailed exposure records (over 100 exposure incidents) obtained through the LEAD framework into a single-page summary for clinicians, since in many cases, clinicians do not have time to review a Veteran’s entire military/exposure history to generate meaningful insights since treatment of acute outcomes like pain take priority. Thus, a systematic method to aggregate a Veteran’s prior exposure data and generate clinically relevant summaries will help keep the focus on the patient’s immediate clinical need while also considering their past exposures.

The three databases presented in this report are integral exposure resources for the reasons detailed in Table 1. However, there are limitations to each of these resources; for this reason, it is important to integrate and combine information from all three of these sources.

EODIMS records operational incidents with potential exposures, some immediate outcomes, occupational duties, and deployment data. However, due to the classified nature of the data, extensive redaction is necessary before exporting data for healthcare use in VA facilities. The LEAD framework streamlines extraction of non-operational health information for exposure assessment and care at VA clinics. While the examples provided here have a focus on EOD related exposures, the methods detailed in the report can generalize across exposures from other military occupations and civilian settings.

VMOAT is a detailed questionnaire that takes around 45 min to complete. It is not meant to be used as a screener but rather designed for Veterans with more complex exposure histories. While the VMOAT provides valuable data on exposures, it is a subjective questionnaire subject to the limitations of recall biases. Integrating VMOAT information with other VA and DoD records into a cohesive format also requires extensive exposure training. The LEAD offers a potential solution for incorporating VMOAT findings with existing exposure databases.

ILER integrates individual and population-level exposure data from VA and DoD databases. However, its reports are extensive which makes it difficult for non-occupational medicine professionals to understand. Information prioritization is not a key focus area, making clinically relevant information extraction time consuming. Additionally, most of the information in ILER is population-level data which may not reflect individual service members’ exposures. Therefore, other sources like EODIMS and VMOAT are needed to identify potential individual level exposures. LEAD enables a holistic framework for how to merge exposure data from multiple sources.

While an expert-informed weighted scoring method can estimate dose, empirical weight assignment using health outcomes is needed for evidence-based risk estimation. It is important to note while interpreting exposures, that exposure-based metrics typically reflects exposure-dose, whereas outcome-based risk estimates reflect exposure toxicity. Additionally, self-report service dates may not reflect official service records (DD214 form). We hope to mitigate this issue by adding both records when available and prioritizing self-reports where available since Veterans many face a variety of barriers to go the appropriate administrative processes to update records. Another inherent limitation is that exposure data can vary across military branches given the unique mandates specific to each branch. To address this aspect, aggregate statistics and sparsity information based on post-hoc analysis specific to each branch of service or units could be reported to help provide context to the exposure profile. Future iterations of LEAD will aim to integrate VA and DoD health records to provide data-driven risk scores to include exposure toxicity in addition to exposure dosage.

## Conclusion

To address Veteran exposure concerns, the VA should collaborate with the DoD and other partners to improve models of how military occupational exposures impact health. This can be achieved by using subject matter expertise and reviewing literature in occupational and environmental medicine. The LEAD framework defines exposure common data elements for collecting and extracting exposure information from various databases to create consistent, succinct, insightful, comprehensive, and clinically relevant exposure profiles. Incorporating these elements in future studies ensures consistency, comparability, and robustness in data collection and analysis. Additionally, the LEAD framework aligns with the PACT ACT directives to understand how hazardous exposures affect Veteran health and helps identify new presumptive conditions for care and benefits. The long-term goal of the LEAD framework is to inform clinically relevant exposure summaries utilizing multiple data sources to optimize clinical and research processes associated with exposure data acquisition and use.

## Data availability statement

The original contributions presented in the study are included in the article/Supplementary material, further inquiries can be directed to the corresponding author.

## Author contributions

IS: Writing – original draft. KP: Writing – review & editing, Data curation. ST: Writing – review & editing, Data curation. MP: Writing – review & editing. CL: Writing – review & editing. AP: Writing – review & editing, Data curation. JM: Writing – review & editing, Data curation. JL: Writing – review & editing, Data curation. TD: Writing – review & editing, Data curation. RF: Writing – review & editing. JO: Writing – review & editing. JB: Writing – review & editing. MR: Writing – review & editing. MC: Writing – review & editing.

## References

[1] TES, V. A. (2023). VA has screened 5 million veterans for toxic exposures, paving the way for early detection and treatment of health conditions VA Wilmington health care veterans affairs. Available at: https://www.va.gov/wilmington-health-care/news-releases/va-has-screened-5-millionveterans-for-toxic-exposures-paving-the-way-for-early-detection-and-treatment-of/

[2] Chung MK Rappaport SM Wheelock CE Nguyen VK van der Meer TP Miller GW. Utilizing a biology-driven approach to map the Exposome in health and disease: an 9 essential investment to drive the next generation of environmental discovery. Environ Health Perspect. (2021) 129:85001. doi: 10.1289/EHP8327, PMID: 34435882 PMC8388254

[3] Stingone JA Buck Louis GM Nakayama SF Vermeulen RC Kwok RK Cui Y. Toward greater implementation of the Exposome research paradigm within environmental epidemiology. Annu Rev Public Health. (2017) 38:315–27. doi: 10.1146/annurev-publhealth-082516-012750, PMID: 28125387 PMC5664945

[4] Lauriola P Crabbe H Behbod B Yip F Medina S Semenza JC. Advancing global health through environmental and public health tracking. Int J Environ Res Public Health. (2020) 17:1976. doi: 10.3390/ijerph17061976, PMID: 32192215 PMC7142667

[5] Thacker SB Stroup DF Parrish RG Anderson HA. Surveillance in environmental public health: issues, systems, and sources. Am J Public Health. (1996) 86:633–8. doi: 10.2105/AJPH.86.5.633, PMID: 8629712 PMC1380469

[6] Hall AL Batchelor T Bogaert L Buckland R Cowieson AB Drew M. International perspective on military exposure data sources, applications, and opportunities for collaboration. Front Public Health. (2023) 11:1154595. doi: 10.3389/fpubh.2023.1154595, PMID: 37213639 PMC10198376

[7] Saunders PJ Middleton JD Rudge G. Environmental public health tracking: a cost-effective system for characterizing the sources, distribution and public health impacts of environmental hazards. J Public Health. (2017) 39:506–13. doi: 10.1093/pubmed/fdw130, PMID: 27908973 PMC5896603

[8] Lai H Levitt BB. The roles of intensity, exposure duration, and modulation on the biological e ects of radiofrequency radiation and exposure guidelines. Electromagn Biol Med. (2022) 41:230255:230–55. doi: 10.1080/15368378.2022.2065683 35438055

[9] Di Credico G Polesel J Dal Maso L Pauli F Torelli N Luce D. Alcohol drinking and head and neck cancer risk: the joint effect of intensity and duration. Br J Cancer. (2020) 123:1456–63. doi: 10.1038/s41416-020-01031-z, PMID: 32830199 PMC7592048

[10] Ouellet S Philippens M. The multi-modal responses of a physical head model subjected to various blast exposure conditions. Shock Waves. (2017) 28:19–36. doi: 10.1007/s00193-017-0771-3

[11] Taylor PA Ford CC. Simulation of blast-induced early-time intracranial wave physics leading to traumatic brain injury. J Biomech Eng. (2009) 131:061007. doi: 10.1115/1.3118765, PMID: 19449961

[12] Barrett J. Samuel I. Breneman C. Prisco M. Costanzo M. Krahl P (2022). V MOAT pilot: the comprehensive veteran-military occupational exposure assessment tool. American Occupational Health Conference.

[13] Samuel I. Pollin K. U. Breneman C. B. Chun T. Valmas M. M. Brewster R. C.. (2022). Effects of military occupational exposures on home-based assessment of veterans self reported health, sleep and cognitive performance measures. International conference on human-computer interaction, 91–102.

[14] VA Public Health. (2023). Individual longitudinal exposure record, an individual, electronic record of exposures designed in collaboration between VA and the Department of Defense (DoD) for each service member and future veteran. Available at: https://www.publichealth.va.gov/exposures/publications/ military-exposures/meyh-1/ILER.asp

[15] OSHA. (2023). Occupational safety and health administration Hazard Identifcation and assessment-Hazard categories. Available at: https://www.osha.gov/safety-management/hazard-Identification#:~:text=Health%20hazards%20include%20chemical%20hazards,%2C%20repetitive%20motions%2C%20vibrationere

[16] Seidman H Seliko IJ Hammond EC. Mortality of brain tumors among asbestos insulation workers in the United States and Canada. Ann N Y Acad Sci. (1982) 381:160171:160–71. doi: 10.1111/j.1749-6632.1982.tb50380.x 6953786

[17] Selikoff IJ Hammond EC Seidman H. Latency of asbestos disease among insulation workers in the United States and Canada. Cancer. (1980) 46:2736–40. doi: 10.1002/1097-0142(19801215)46:12<2736::AID-CNCR2820461233>3.0.CO;2-L 7448712

[18] CDC. (2022). Incidence of malignant mesothelioma, 19992018 CDC. Available at: https://www.cdc. gov/cancer/uscs/about/data-briefs/no27-incidence-malignant-mesothelioma-1999-2018.htm

[19] Kaufman LR LeMasters GK Olsen DM Succop P. E ects of concurrent noise and jet fuel exposure on hearing loss. J Occup Environ Med. (2005) 47:212–8. doi: 10.1097/01.jom.0000155710.28289.0e 15761316

[20] Ankley GT Cureton P Hoke RA Houde M Kumar A Kurias J. Assessing the ecological risks of per- and Polyfluoroalkyl substances: current state-of-the science and a proposed path forward. Environ Toxicol Chem. (2020) 40:564–605. doi: 10.1002/etc.4869 32897586 PMC7984443

[21] Seals R Bartell SM Steenland K. Accumulation and clearance of per uorooctanoic acid (PFOA) in current and former residents of an exposed community. Environ Health Perspect. (2011) 119:119–24. doi: 10.1289/ehp.1002346, PMID: 20870569 PMC3018490

[22] Wang Z Wilson CM Mendelev N Ge Y Galfalvy H Elder G. Acute and chronic molecular signatures and associated symptoms of blast exposure in military Breachers. J Neurotrauma. (2020) 37:1221–32. doi: 10.1089/neu.2019.6742, PMID: 31621494 PMC7232647

[23] Hymel PA Loeppke RR Baase CM Burton WN Hartenbaum NP Hudson TW. Workplace health protection and promotion: a new pathway for a healthier and safer workforce. J Occup Environ Med. (2011) 53:695702:695–702. doi: 10.1097/JOM.0b013e31822005d0 21654443

[24] Larson GE Highfill-McRoy RM Booth-Kewley S. Psychiatric diagnoses in historic and contemporary military cohorts: combat deployment and the healthy warrior effect. Am J Epidemiol. (2008) 167:1269–76. doi: 10.1093/aje/kwn084, PMID: 18436536

[25] Siroux V Agier L Slama R. The exposome concept: a challenge and a potential driver for environmental health research. Eur Respir Rev. (2016) 25:124129:124–9. doi: 10.1183/16000617.0034-2016 PMC9487242 27246588

[26] Vermeulen R Schymanski EL Barabasi A-L Miller GW. The exposome and health: where chemistry meets biology. Science. (2020) 367:392396:392–6. doi: 10.1126/science.aay3164 PMC7227413 31974245

[27] NIOSH. (2024). Hierarchy of controls, the National Institute for Occupational Safety and Health (NIOSH), Centers for Disease Control and Prevention. Available at: https://www.cdc.gov/niosh/topics/ hierarchy/default.html

[28] Reddy SC Valderrama AL Kuhar DT. Improving the use of personal protective equipment: applying lessons learned. Clin Infect Dis. (2019) 69:S165–70. doi: 10.1093/cid/ciz619 31517978

