# Supplementary material for: Linked Exposures Across Databases: an exposure common data elements aggregation framework to facilitate clinical exposure review
Source: Front Public Health. 2024 Jun 28;12:1408222. doi: 10.3389/fpubh.2024.1408222 (PMC11243485; doi:10.3389/fpubh.2024.1408222)
Supplement: Supplementary file 1 [file Data_Sheet_1.docx]

Supplementary Material

# Supplementary Tables

**Supplementary Table 1.** Sample high / low exposure data extracted using the LEAD framework shows available data for a specific exposure type (blast) for two mock EOD Veterans. Exposures reported are not tied to an individual but pulled from representative sources to provide an example of how the LEAD framework can be used to compare differences in exposure dose for a specific exposure.

| **S1. Blast Exposure Collation** | | **Intensity** | | | **Time** | | | **Moderators** |
| --- | --- | --- | --- | --- | --- | --- | --- | --- |
| **Source** | **Exposure** | **Route** | **Proximity** | **Symptoms** | **Period** | **Duration** | **Frequency** | **Protective Controls** |
| Low Exposed |  | | | | | | | |
| EODIMS | Blast | Skin Contact | 250 feet | None | 03/01/1999 – 03/02/1999 | 4 hours | 1 event | Suit |
| VMOAT | Blast | Skin Contact (dust) | within 500 feet | None | 05/01/1985 – 03/02/1999 | 4 hours | Infrequent (once a year or less) | Gloves, Eyewear, Headgear |
| ILER-PDHA | Blast or Explostion (land mine, grenade,etc.) | Impact | More than 100 meters (328 feet) | None | 03/01/1999 – 03/02/1999 | n/a | 1 event | n/a |
| High Exposed |  | | | | | | | |
| EODIMS | Blast | Impact, Inhalation | 25 feet | Lost consciousness | 03/01/1999 – 03/02/1999 | 4 hours | 1 event | Suit |
| VMOAT | Blast | Impact, Inhalation, Skin Contact, Eye Contact | within 50 feet | Severe (Lost consciousness 30minutes-24 hours) | 01/01/1984 – 10/01/1994 | 2-4 hours per day | Frequent (Daily) | None |
| ILER-PDHA | Blast or Explosion (land mine, grenade, etc.) | Impact (not directly listed) | Less than 25 meters (82 feet) | LOC 5-30 min, Seeing stars | 03/01/1999 – 03/02/1999 | n/a | more than 3 (5 times) | n/a |

**Supplementary Table 2.** Score card used to translate exposure information in table S1 to categorical scores as shown in table 4.

| **S2. Scoring** | **Intensity** | | | **Time** | | | **Moderators** |
| --- | --- | --- | --- | --- | --- | --- | --- |
| **Score** | **Route** | **Proximity** | **Symptoms** | **Period** | **Duration** | **Frequency** | **Protective Controls** |
| 0 | na | na | none | na | na | na | none |
| 1 | 1 route | >250m | 1 Very Mild | 1 day - 1 week | < 1 hour | 1 event | 1 |
| 2 | 2 routes | 100m - 250m | 2 Mild | 1 week - 1 month | 1-2 hours | 2 events | 2 |
| 3 | 3 routes | 10m - 100m | 3 Moderate | 1 month - 1 year | 2-4 hours | 3 events | 3 |
| 4 | 4 routes | 1m - 10m | 4 Severe | 1 year - 10 years | 4-8 hours | >4 events | 4 |
| 5 | >5 routes | <1m | 5 Very Severe | >10 years | >8 hours | routine exposure | >5 |

**Supplementary Table 3.** Dose Estimation: A sample scoring of blast exposure data across multiple exposure databases for two mock EOD Veterans. The scoring is based on weights assigned by exposure experts meant to represent the clinical significance of the exposure data in table S1. Note: Data on individual differences is sparse at present, so this example is representative of the types of information that are currently available. Future efforts to estimate individual differences data from other health assessment systems is needed since that is an important aspect of exposure dose estimation.

| S3. Dose Estimation | **Variables** | | | | | | | |
| --- | --- | --- | --- | --- | --- | --- | --- | --- |
|  | **Route** | **Proximity** | **Symptoms** | **Period** | **Duration** | **Frequency** | **Protective Controls** | **Weighted Score** |
| Expert Informed Weights | 1 | 1 | 5 | 1 | 2 | 2 | -2 |  |
|  |  |  |  |  |  |  |  |  |
| Blast Exposure Data |  |  |  |  |  |  |  |  |
| Low Exposed Example |  |  |  |  |  |  |  |  |
| EODIMS | 1 | 1 | 0 | 1 | 3 | 1 | 1 | 16 |
| VMOAT | 1 | 1 | 0 | 4 | 3 | 1 | 3 | 18 |
| ILER-PDHA | 1 | 2 | 0 | 1 |  | 1 |  | 9 |
| Average: | | | | | | | | 14.33 |
| High Exposed Example |  |  |  |  |  |  |  |  |
| EODIMS | 2 | 4 | 3 | 1 | 3 | 1 | 1 | 187 |
| VMOAT | 4 | 3 | 4 | 4 | 3 | 5 | 0 | 540 |
| ILER-PDHA | 1 | 4 | 2 | 1 |  | 4 |  | 135 |
| Average: | | | | | | | | 287.33 |
